# Bioinformatic analysis of beta carbonic anhydrase sequences from protozoans and metazoans

**DOI:** 10.1186/1756-3305-7-38

**Published:** 2014-01-21

**Authors:** Reza Zolfaghari Emameh, Harlan Barker, Martti E E Tolvanen, Csaba Ortutay, Seppo Parkkila

**Affiliations:** 1 School of Medicine, University of Tampere, Medisiinarinkatu 3, 33520 Tampere, Finland; 2 Institute of Biomedical Technology and BioMediTech, University of Tampere, 33520 Tampere, Finland; 3 Department of Information Technology, University of Turku, 20520 Turku, Finland; 4 Fimlab Ltd and Tampere University Hospital, Biokatu 4, 33520 Tampere, Finland

**Keywords:** Beta carbonic anhydrase, Inhibitor, Metazoa, Mitochondrial targeting peptide, Multiple sequence alignment, Protozoa

## Abstract

**Background:**

Despite the high prevalence of parasitic infections, and their impact on global health and economy, the number of drugs available to treat them is extremely limited. As a result, the potential consequences of large-scale resistance to any existing drugs are a major concern. A number of recent investigations have focused on the effects of potential chemical inhibitors on bacterial and fungal carbonic anhydrases. Among the five classes of carbonic anhydrases (alpha, beta, gamma, delta and zeta), beta carbonic anhydrases have been reported in most species of bacteria, yeasts, algae, plants, and particular invertebrates (nematodes and insects). To date, there has been a lack of knowledge on the expression and molecular structure of beta carbonic anhydrases in metazoan (nematodes and arthropods) and protozoan species.

**Methods:**

Here, the identification of novel beta carbonic anhydrases was based on the presence of the highly-conserved amino acid sequence patterns of the active site. A phylogenetic tree was constructed based on codon-aligned DNA sequences. Subcellular localization prediction for each identified invertebrate beta carbonic anhydrase was performed using the TargetP webserver.

**Results:**

We verified a total of 75 beta carbonic anhydrase sequences in metazoan and protozoan species by proteome-wide searches and multiple sequence alignment. Of these, 52 were novel, and contained highly conserved amino acid residues, which are inferred to form the active site in beta carbonic anhydrases. Mitochondrial targeting peptide analysis revealed that 31 enzymes are predicted with mitochondrial localization; one was predicted to be a secretory enzyme, and the other 43 were predicted to have other undefined cellular localizations.

**Conclusions:**

These investigations identified 75 beta carbonic anhydrases in metazoan and protozoan species, and among them there were 52 novel sequences that were not previously annotated as beta carbonic anhydrases. Our results will not only change the current information in proteomics and genomics databases, but will also suggest novel targets for drugs against parasites.

## Background

Carbonic anhydrases (CAs) are ubiquitous metalloenzymes. They are encoded by five evolutionary divergent gene families and the corresponding enzymes are designated α, β, γ, δ and ζ-CAs. α-CAs are present in animals, some fungi, bacteria, algae, and cytoplasm of green plants. β-CAs are expressed mainly in fungi, bacteria, archaea, algae, and chloroplasts of monocotyledons and dicotyledons. γ-CAs are expressed in plants, archaea, and some bacteria. δ- and ζ-CAs are present in several classes of marine phytoplankton [1-6]. A total of 13 enzymatically active α-CAs have been reported in mammals: CA I, CA II, CA III, CA VII, and CA XIII are cytosolic enzymes; CA IV, CA IX, CA XII, CA XIV, and CA XV are membrane-bound; CA VA and CA VB are mitochondrial; CA VI is secreted and CA VIII, CA X, and CA XI are acatalytic CA-related proteins [3,7]. The active site of CA contains a zinc ion (Zn^2+^) which has a critical role in the catalytic activity of the enzyme. ζ-and γ-CAs represent exceptions to this rule since they can use cadmium (ζ), iron (γ), or cobalt (γ) as cofactors [8-10]. CAs are involved in many biological processes, such as respiration involving transport of CO_2_ and bicarbonate between metabolizing tissues, pH homeostasis, electrolyte transfer, bone resorption, calcification, and tumor progression. They also participate in some biosynthetic reactions, such as gluconeogenesis, lipogenesis, and ureagenesis [3,11-14].

The first β-CA was serendipitously discovered by Neish in 1939 [15]. In 1990, the cDNA sequence of spinach (*Spinacea oleracea*) chloroplast CA was determined, and found to be non-homologous to animal α-CA [16,17]. Thereafter, cDNA sequences of β-CA from pea (*Pisium sativum*) and *Arabidopsis thaliana* were determined [17-19]. It is believed that the plant β-CAs are distributed in the chloroplastic stroma, thylakoid space, and cytoplasm of plant cells [17]. Many putative β-CAs have been discovered since 1990, not only in photosynthetic organisms, but also in eubacteria, yeast, and archaea [17].

The first bacterial β-CA gene was named *CynT* and recognized in *Escherichia coli*[20,21]. Later, β-CA was identified in some other pathogenic bacteria, such as *Helicobacter pylori, Mycobacterium tuberculosis, Salmonella typhimurium*[17,22], *Haemophilus influenzae*[23,24], *Brucella suis*[24,25], *Streptococcus pneumoniae*[24,26], *Salmonella enterica*[24,27], and *Vibrio cholerae*[24,28,29]. β-CAs have also been identified in fungi, such as *Candida albicans*[1,30], *Candida glabrata*[1,31], *Cryptococcus neoformans*[1,32], and *Sordaria macrospora*[6,33]. This class of enzyme has also been discovered in a wide range of taxa, such as yeast (*Saccharomyces cerevisiae*) [34-36], cyanobacteria (*Synechocystis* sp. PCC6803) [37], carboxysomes of chemoautotrophic bacteria (*Halothiobacillus neapolitanus*) [38], green algae (*Chlamydomonas reinhardtii*) [39], red algae (*Porphyridium purpureum*) [40], nematodes (*Caenorhabditis elegans)*[41], and insects (*Drosophila melanogaster)*[4]. While β-CAs were initially thought to be expressed only in plants, this enzyme family is indeed present in a wide variety of species – from bacteria and archaea to invertebrate animals, missing only from vertebrates and most chordates, making it an attractive target for evolutionary studies [5].

β-CA is an important accessory enzyme for many CO_2_ or HCO_3_^-^ utilizing enzymes (e.g. RuBisCO in chloroplasts, cyanase in *E. coli*[42], urease in *H. pylori*[43], and carboxylases in *Corynebacterium glutamicum*[44]). In cyanobacteria, β-CA is an essential component of the CO_2_-concentrating carboxysome organelle [17,45]. β-CA activity is required for growth of *E. coli* bacteria in air [46]; it is also indispensable if the atmospheric partial pressure of CO_2_ is high or during anaerobic growth in a closed vessel at low pH, where copious CO_2_ is generated endogenously. β-CA is also needed for growth of *C. glutamicum*[44,47] and some yeasts, such as *S. cerevisiae*[40]. In higher plants, the *Flaveria bidentis* genome contains at least three β-CA genes, named *CA1*, *CA2*, and *CA3*[48]. The functional roles of β-CAs in plants are not yet fully understood, even though a lot of new data has emerged in recent years. C_3_ and C_4_ plants have different mechanisms for carbon fixation and photosynthesis and, thus, β-CAs might possess different roles, depending on the location of the enzyme and the type of plant [49]. In plants, the highest CA activity has been found within the chloroplast stroma, but there is also some CA activity in the cytosol of mesophyll cells [50]. Carbon dioxide coming from the external environment must be rapidly hydrated by β-CA and converted into HCO_3_^−^ for the phosphoenolpyruvate carboxylase enzyme [49]. Additionally, CAs play a role in photosynthesis by facilitating diffusion into and across the chloroplast, and by catalyzing HCO_3_^-^ dehydration to supply CO_2_ for RuBisCO. Interestingly, both RuBisCO and β-CA expression levels increase together when *P. sativum* is transferred from an environment with high levels of CO_2_ to one with low levels [47].

Crystal structures of β-CAs reveal that a zinc ion (Zn^2+^) is ligated by two conserved cysteines and one conserved histidine [5]. Until now, the only X-ray crystallography structure defined for β-CAs in plants belongs to *P. sativum*[51]. *E. coli* was the first bacteria in which the β-CA crystal structure was determined [20]. β-CA can adopt a variety of oligomeric states with molecular masses ranging from 45 to 200 kDa [52].

The first metazoan β-CAs were reported in 2010 [41]. In one of the studies [4,41], two genes encoding β-CAs (y116a8c.28 and bca-1) were identified in *Caenorhabditis elegans*. Another study reported a novel β-CA gene identified from FlyBase, which was named DmBCA (short for *Drosophila melanogaster* β-CA) [4]. Additionally, orthologs were retrieved from sequence databases, and reconstructed when necessary. The results confirmed the presence of β-CA sequences in 55 metazoan species, such as *Aedes aegypti, Culex quinquefasciatus, Anopheles gambiae, Drosophila virilis, Tribolium castaneum, Nasonia vitripennis, Apis mellifera, Acyrthosiphon pisum, Daphnia pulex, Caenorhabditis elegans, Pristionchus pacificus, Trichoplax adhaerens, Caligus clemensi, Lepeophtheirus salmonis, Nematostella vectensis, Strongylocentrotus purpuratus,* and *Saccoglossus kowalevskii*. The DmBCA enzyme was produced as a recombinant protein in Sf9 insect cells, and its kinetic and inhibition profiles were determined. The enzyme showed high CO_2_ hydratase activity, with a k_cat_ of 9.5 × 10^5^ s^-1^ and a k_cat_/K_M_ of 1.1 × 10^8^ M^-1^ s^-1^. DmBCA was inhibited by the clinically-used sulfonamide, acetazolamide, with an inhibition constant of 49 nM. Subcellular localization studies have indicated that DmBCA is probably a mitochondrial enzyme, as is also suggested by sequence analysis.

In this study, using bioinformatics tools, we discovered and verified the presence of β-CA in various other metazoan species, and, for the first time, in protozoa. Previously, most β-CA proteins have been identified in protein databases as ‘unknown’ proteins or ‘putative’ CAs, without a specific reference to β-CAs. Based on the present findings, new avenues will be opened to biochemically characterize β-CAs and their inhibitors in arthropods, nematodes and protozoans.

## Methods

### Identification of putative β-CA enzymes in protozoan and metazoan species and multiple sequence alignment

Identification of novel β-CAs was based on the presence of the highly-conserved amino acid sequence patterns of the active site, namely Cys-Xaa-Asp-Xaa-Arg and His-Xaa-Xaa-Cys also marked in Additional file 1: Figure S1. Alignment was visualized in Jalview [53]. In total, 75 invertebrate β-CA sequences were retrieved from Uniprot (http://www.uniprot.org/) for alignment analysis, and one bacterial sequence (*Pelosinus fermentans*) was included as an outgroup. All protein sequences were aligned using Clustal Omega (http://www.ebi.ac.uk/Tools/msa/clustalo/) [54]. The sequences were manually curated to remove residues associated with an incorrect starting methionine. A total of 90 residues were removed from the N-terminal end of Uniprot IDs D4NWE5_ADIVA, G0QPN9_ICHMG, D6WK56_TRICA, I7LWM1_TETTS and I7M0M0_TETTS. The modified protein sequences were then re-aligned. This protein alignment then served as the template for codon alignment of corresponding nucleotide sequences using the Pal2Nal program (http://www.bork.embl.de/pal2nal/) [55].

### Phylogenetic analysis

The phylogenetic analysis was computed using Mr. Bayes v3.2 [56]. After 8 million generations using the GTR codon substitution model, with all other parameters as default, the standard deviation of split frequencies was 1.39 × 10^-3^. The final output tree was produced using 50% majority rule consensus. FigTree v1.4.0 (http://tree.bio.ed.ac.uk/software/figtree/) [56] was used to visualize the phylogenetic tree and the *Pelosinus fermentans*[57] sequence set as outgroup. Additional trees were constructed for comparison using maximum likelihood (PhyML)[58], UPGMA, and neighbor-joining methods within Geneious version 7.0.5 from Biomatters (Auckland, New Zealand) (http://www.geneious.com/).

### Prediction of subcellular localization

Subcellular localization prediction of each identified invertebrate β-CA was performed using the TargetP webserver (http://www.cbs.dtu.dk/services/TargetP/). TargetP is built from two layers of neural networks, where the first layer contains one dedicated network for each type of pre-sequence [cTP (cytoplasmic targeting peptide), mTP (mitochondrial targeting peptide, or SP (secretory signal peptide)], and the second is an integrating network that outputs the actual prediction (cTP, mTP, SP, other). It is able to discriminate between cTPs, mTPs, and SPs with sensitivities and specificities higher than what has been obtained with other available subcellular localization predictors [59].

## Results

### Multiple sequence alignment

The Uniprot search of potential β-CA sequences, and the subsequent multiple sequence alignment, identified 75 β-CAs in metazoan and protozoan species, of which 23 sequences were reported as β-CAs previously [4]. Thus, 52 metazoan and protozoan β-CA sequences were novel and reported here for the first time. All 75 β-CAs in metazoan and protozoan species are shown in Table 1. The multiple sequence alignment results of these 75 β-CAs, plus a bacterial β-CA sequence from *Pelosinus fermentans*, are shown as Additional file 1: Figure S1. Multiple sequence alignment of all animal β-CAs confirmed conservation of the known active site motifs CxDxR and HxxC in all identified enzymes. Several other key residues were also highly conserved. Notably, all β-CA sequences from *Leishmania* species (*Leishmania donovani*, *Leishmania infantum*, *Leishmania major*, and *Leishmania mexicana*) contained a 71 residue N-terminal extension not present in any other sequences.

**Table 1:** Identified β-CAs in protozoan and metazoan species

**Species**	**β- CA ID**	**Entry ID**	**Gene name**	**Protein name**
***Acromyrmex echinatior***	BCA	F4WAG3	G5I_02499	Beta carbonic anhydrase 1
***Acyrthosiphon pisum***	BCA1	J9K706	Uncharacterized	Uncharacterized
	BCA2	C4WVD8	ACYPI006033	ACYPI006033
	BCA3	J9JZY3	XM_001950078.1	Uncharacterized
***Adineta vaga***	BCA	D4NWE5	Uncharacterized	Putative uncharacterized protein
***Aedes aegypti***	BCA	Q17N64	AAEL000816	AAEL000816-PA
***Ancylostoma caninum***	BCA	FC551456	Uncharacterized	Uncharacterized protein
***Anopheles darlingi***	BCA	E3X5Q8	AND_14274	Uncharacterized protein
***Anopheles gambiae***	BCA	Q5TU56	AGAP002992 AgaP_AGAP002992	AGAP002992-PA
***Apis mellifera***	BCA	H9KS29	Uncharacterized	Uncharacterized protein
***Ascaris suum***	BCA	F1LE18	Uncharacterized	Beta carbonic anhydrase 1
***Caenorhabditis brenneri***	BCA1	G0MSW4	Cbn-bca-1 CAEBREN_17105	CBN-BCA-1 protein
	BCA2	G0MRG1	Cbn-bca-2 CAEBREN_06024	CBN-BCA-2 protein
***Caenorhabditis briggsae***	BCA1	A8XKV0	bca-1 CBG14861	Beta carbonic anhydrase 1
	BCA2	A8WN21	bca-2 Cbr-bca-2 cbr-bca-2 CBG00424 CBG_00424	Protein CBR-BCA-2
***Caenorhabditis elegans***	BCA1	Q22460	bca-1 T13C5.5	Beta carbonic anhydrase 1
	BCA2	Q2YS41	bca-2 Y116A8C.28	Protein BCA-2
***Caenorhabditis remanei***	BCA1	E3LDN3	Cre-bca-1 CRE_00190	CRE-BCA-1 protein
	BCA2	E3MK96	Cre-bca-2 CRE_28742	CRE-BCA-2 protein
***Caligus clemensi***	BCA	C1C2M7	CYNT	Carbonic anhydrase
***Camponotus floridanus***	BCA	E2ANQ9	EAG_05651	Carbonic anhydrase
***Culex quinquefasciatus***	BCA	B0WKV7	CpipJ_CPIJ007527	Carbonic anhydrase
***Danaus plexippus***	BCA	G6D7Z4	Uncharacterized	Putative carbonic anhydrase
***Daphnia pulex***	BCA	E9GLB5	CAB	Beta-carbonic anhydrase
***Dendroctonus ponderosae***	BCA	J3JTM9	Uncharacterized	Uncharacterized protein
***Drosophila ananassae***	BCA	B3LZ10	GF17694 Dana\GF17694 Dana_GF17694	GF17694
***Drosophila erecta***	BCA	B3P1V8	GG13874 Dere\GG13874 Dere_GG13874	GG13874
***Drosophila grimshawi***	BCA	B4JHY1	GH19010 Dgri\GH19010 Dgri_GH19010	GH19010
***Drosophila melanogaster***	BCA	Q9VHJ5	CAHbeta CG11967 Dmel_CG11967	CG11967
***Drosophila mojavensis***	BCA	B4KDC1	GI23065 Dmoj\GI23065 Dmoj_GI23065	GI23065
***Drosophila persimilis***	BCA	B4GFA1	GL22171 Dper\GL22171 Dper_GL22171	GL22171
***Drosophila pseudoobscura***	BCA	Q296E4	GA11301 Dpse\GA11301 Dpse_GA11301	GA11301
***Drosophila sechellia***	BCA	B4HKY7	GM23772 Dsec\GM23772 Dsec_GM23772	GM23772
***Drosophila simulans***	BCA	B4QXC5	GD18582 Dsim\GD18582 Dsim_GD18582	GD18582
***Drosophila virilis***	BCA	B4LZE7	CAHbeta Dvir\GJ24578 GJ24578 Dvir_GJ24578	GJ24578
***Drosophila willistoni***	BCA	B4NBB9	GK11865 Dwil\GK11865 Dwil_GK11865	GK11865
***Drosophila yakuba***	BCA	B4PTY0	GE25916 Dyak\GE25916 Dyak_GE25916	GE25916
***Entamoeba dispar***	BCA	B0E7M0	EDI_275880	Carbonic anhydrase
***Entamoeba histolytica***	BCA	C4LXK3	EHI_073380	Carbonic anhydrase
***Entamoeba nuttalli***	BCA	K2GQM0	ENU1_204230	Carbonate dehydratase domain containing protein
***Harpegnathos saltator***	BCA	E2B2Q1	EAI_05019	Carbonic anhydrase
***Heliconius melpomene***	BCA	HMEL015257	Uncharacterized	Uncharacterized protein
***Hirudo medicinalis***	BCA	EY481200	Uncharacterized	Uncharacterized protein
***Ichthyophthirius multifiliis***	BCA	G0QPN9	IMG5_069900	Carbonic anhydrase
***Leishmania donovani***	BCA	E9B8S3	LDBPK_060630	Carbonic anhydrase
***Leishmania infantum***	BCA	A4HSV2	LINJ_06_0630	Carbonic anhydrase
***Leishmania major***	BCA	Q4QJ17	LMJF_06_0610	Carbonic anhydrase
***Leishmania mexicana***	BCA	E9AKU0	LMXM_06_0610	Carbonic anhydrase
***Lepeophtheirus salmonis***	BCA	D3PI48	BCA1	Beta carbonic anhydrase 1
***Nasonia vitripennis***	BCA	K7IWK8	Uncharacterized	Uncharacterized protein
***Nematostella vectensis***	BCA	A7S717	v1g186479	Predicted protein
***Paramecium tetraurelia***	BCA1	A0BD61	GSPATT00004572001	Carbonic anhydrase
	BCA2	A0E8J0	GSPATT00024336001	Carbonic anhydrase
	BCA3	A0CEX6	GSPATT00037782001	Carbonic anhydrase
	BCA4	A0BDB1	GSPATT00004622001	Carbonic anhydrase
	BCA5	A0C922	GSPATT00006595001	Carbonic anhydrase
***Saccoglossus kowalevskii***	BCA	187043763	Uncharacterized	Uncharacterized protein
***Schistosoma mansoni***	BCA	G4V6B2	Smp_004070	Putative carbonic anhydrase
***Solenopsis invicta***	BCA	E9IP13	SINV_09652	Putative carbonic anhydrase
***Strigamia maritima***	BCA	SMAR006741	Uncharacterized	Uncharacterized protein
***Strongylocentrotus purpuratus***	BCA	H3I177	Uncharacterized	Uncharacterized protein
***Tetrahymena thermophila***	BCA1	Q22U21	TTHERM_00263620	Carbonic anhydrase
	BCA2	Q22U16	TTHERM_00263670	Carbonic anhydrase
	BCA3	I7MDL7	TTHERM_00373840	Carbonic anhydrase
	BCA4	I7LWM1	TTHERM_00558270	Carbonic anhydrase
	BCA5	I7M0M0	TTHERM_00374880	Carbonic anhydrase
	BCA6	I7MD92	TTHERM_00541480	Carbonic anhydrase
	BCA7	I7M748	TTHERM_00374870	Carbonic anhydrase
	BCA8	Q23AV1	TTHERM_00654260	Carbonic anhydrase
***Tribolium castaneum***	BCA	D6WK56	TcasGA2_TC014816	Putative uncharacterized protein
***Trichinella spiralis***	BCA	E5SH53	Uncharacterized	Carbonic anhydrase
***Trichomonas vaginalis***	BCA1	A2ENQ8	TVAG_005270	Carbonic anhydrase
	BCA2	A2DLG4	TVAG_268150	Carbonic anhydrase
***Trichoplax adhaerens***	BCA	B3S5Y1	TRIADDRAFT_29634	Putative uncharacterized protein
***Xenoturbella bocki***	BCA	117195962	Uncharacterized	Uncharacterized protein

### Phylogenetic analysis

**Figure 1:**
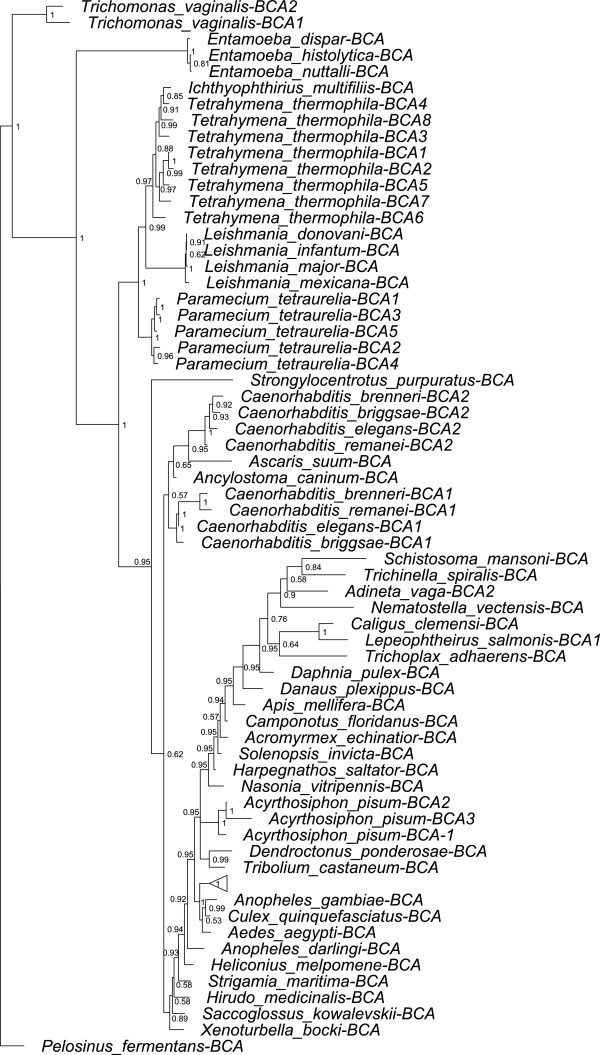
**Phylogenetic analysis of 75 metazoan and protozoan β-CAs.** The position of β-CAs of *Drosophila* species has been represented at the bottom of the phylogenetic tree by a triangle shape. The details of β-CAs of *Drosophila* species in the phylogenetic tree are shown in Figure 2.

**Figure 2:**
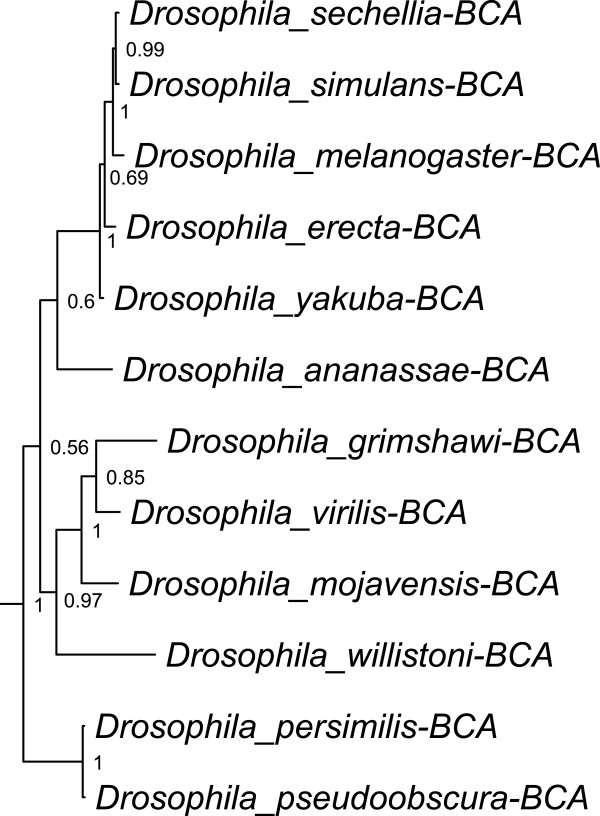
**Phylogenetic analysis of β-CAs of *Drosophila *species.** This tree represents the expanded view of the triangle located near the bottom of the main phylogenetic tree of β-CAs in Figure 1.

The results of the phylogenetic analysis of 75 β-CAs in metazoan and protozoan species are shown in Figure 1. A β-CA sequence from the *Pelosinus fermentans* bacterium was used as an outgroup [60]. The phylogenetic results represent the evolutionary root of β-CAs in metazoan and protozoan species, the similarity between them, and duplications that have occurred. The branching pattern and branch lengths reveal interesting evolutionary relationships of β-CAs in various invertebrate species. There is a close relationship between our bacterial outgroup and *Trichomonas vaginalis* β-CAs, both having originated well before the other species within the tree. β-CAs of nematodes and arthropods are located in the lower evolutionary branches. In the protozoan *Tetrahymena thermophilia* and *Paramecium tetraurelia* clades significant duplications of β-CA have occurred, with 8 and 5 distinct proteins respectively. Meanwhile, metazoan and nematode species tend to have just one or two β-CAs. Surprisingly, β-CAs of the nematode *Trichinella spiralis* and trematode *Schistosoma mansoni* appear more closely related to arthropod than to nematode enzymes. The triangle located near the bottom of Figure 1 represents the clade of β-CAs in different *Drosophila* species. The details of the phylogenetic tree of β-CAs in *Drosophila* species are shown in Figure 2. The likely presence of inaccuracies in some of the database sequences, and inherent limitations of Bayesian inference, prompted use of additional phylogenetic methods. These analyses generally supported the major features of the final tree achieved via Bayesian inference.

### Subcellular localization of β-CAs

The predictions for subcellular localization of the 75 β-CAs are shown in Table 2. The results reveal that 31 are predicted to have a mitochondrial localization, one (*Anopheles darlingi*, Uniprot ID: E3X5Q8) was predicted to be secreted, and the remaining 43 were predicted to have other cellular localizations. The predictions were based on the analysis of 175 N-terminal amino acids of each sequence. In the **Name** column, there are both IDs of the β-CAs in Uniprot database and scientific name of the metazoan and protozoan species.

**Table 2:** Prediction of the subcellular localization of 75 β-CAs of metazoan and protozoan species

**Species**	**β- CA ID**	**Entry ID**	**mTP**	**SP**	**Other**	**Loc**	**RC**
***Acromyrmex echinatior***	BCA	F4WAG3	0.199	0.054	0.86	-	2
***Acyrthosiphon pisum***	BCA1	J9K706	0.473	0.05	0.631	-	5
	BCA2	C4WVD8	0.579	0.043	0.536	M	5
	BCA3	J9JZY3	0.579	0.043	0.534	M	5
***Adineta vaga***	BCA	D4NWE5	0.509	0.102	0.375	M	5
***Aedes aegypti***	BCA	Q17N64	0.589	0.029	0.491	M	5
***Ancylostoma caninum***	BCA	FC551456	0.466	0.046	0.514	-	5
***Anopheles darlingi***	BCA	E3X5Q8	0.044	0.836	0.144	S	2
***Anopheles gambiae***	BCA	Q5TU56	0.713	0.03	0.34	M	4
***Apis mellifera***	BCA	H9KS29	0.126	0.08	0.875	-	2
***Ascaris suum***	BCA	F1LE18	0.388	0.079	0.406	-	5
***Caenorhabditis brenneri***	BCA1	G0MSW4	0.522	0.036	0.518	M	5
	BCA2	G0MRG1	0.52	0.051	0.473	M	5
***Caenorhabditis briggsae***	BCA1	A8XKV0	0.392	0.047	0.615	-	4
	BCA2	A8WN21	0.546	0.048	0.466	M	5
***Caenorhabditis elegans***	BCA1	Q22460	0.475	0.039	0.549	-	5
	BCA2	Q2YS41	0.465	0.05	0.529	-	5
***Caenorhabditis remanei***	BCA1	E3LDN3	0.327	0.045	0.69	-	4
	BCA2	E3MK96	0.51	0.051	0.48	M	5
***Caligus clemensi***	BCA	C1C2M7	0.21	0.04	0.873	-	2
***Camponotus floridanus***	BCA	E2ANQ9	0.325	0.051	0.735	-	3
***Culex quinquefasciatus***	BCA	B0WKV7	0.573	0.032	0.507	M	5
***Danaus plexippus***	BCA	G6D7Z4	0.793	0.032	0.273	M	3
***Daphnia pulex***	BCA	E9GLB5	0.157	0.055	0.843	-	2
***Dendroctonus ponderosae***	BCA	J3JTM9	0.27	0.064	0.742	-	3
***Drosophila ananassae***	BCA	B3LZ10	0.537	0.041	0.518	M	5
***Drosophila erecta***	BCA	B3P1V8	0.531	0.04	0.53	M	5
***Drosophila grimshawi***	BCA	B4JHY1	0.605	0.037	0.454	M	5
***Drosophila melanogaster***	BCA	Q9VHJ5	0.531	0.04	0.53	M	5
***Drosophila mojavensis***	BCA	B4KDC1	0.556	0.039	0.511	M	5
***Drosophila persimilis***	BCA	B4GFA1	0.595	0.037	0.466	M	5
***Drosophila pseudoobscura***	BCA	Q296E4	0.595	0.037	0.466	M	5
***Drosophila sechellia***	BCA	B4HKY7	0.531	0.04	0.53	M	5
***Drosophila simulans***	BCA	B4QXC5	0.531	0.04	0.53	M	5
***Drosophila virilis***	BCA	B4LZE7	0.531	0.04	0.53	M	5
***Drosophila willistoni***	BCA	B4NBB9	0.531	0.04	0.53	M	5
***Drosophila yakuba***	BCA	B4PTY0	0.531	0.04	0.53	M	5
***Entamoeba dispar***	BCA	B0E7M0	0.114	0.158	0.766	-	2
***Entamoeba histolytica***	BCA	C4LXK3	0.113	0.151	0.779	-	2
***Entamoeba nuttalli***	BCA	K2GQM0	0.132	0.142	0.763	-	2
***Harpegnathos saltator***	BCA	E2B2Q1	0.248	0.055	0.801	-	3
***Heliconius melpomene***	BCA	HMEL015257	0.77	0.032	0.302	M	3
***Hirudo medicinalis***	BCA	EY481200	0.121	0.098	0.778	-	2
***Ichthyophthirius multifiliis***	BCA	G0QPN9	0.181	0.04	0.872	-	2
***Leishmania donovani***	BCA	E9B8S3	0.106	0.13	0.826	-	2
***Leishmania infantum***	BCA	A4HSV2	0.106	0.13	0.826	-	2
***Leishmania major***	BCA	Q4QJ17	0.108	0.124	0.822	-	2
***Leishmania mexicana***	BCA	E9AKU0	0.109	0.135	0.82	-	2
***Lepeophtheirus salmonis***	BCA	D3PI48	0.126	0.068	0.889	-	2
***Nasonia vitripennis***	BCA	K7IWK8	0.388	0.046	0.713	-	4
***Nematostella vectensis***	BCA	A7S717	0.775	0.052	0.211	M	3
***Paramecium tetraurelia***	BCA1	A0BD61	0.196	0.045	0.843	-	2
	BCA2	A0E8J0	0.107	0.056	0.909	-	1
	BCA3	A0CEX6	0.28	0.045	0.725	-	3
	BCA4	A0BDB1	0.073	0.065	0.938	-	1
	BCA5	A0C922	0.178	0.056	0.826	-	2
***Saccoglossus kowalevskii***	BCA	187043763	0.565	0.049	0.463	M	5
***Schistosoma mansoni***	BCA	G4V6B2	0.388	0.064	0.605	-	4
***Solenopsis invicta***	BCA	E9IP13	0.326	0.052	0.756	-	3
***Strigamia maritima***	BCA	SMAR006741	0.683	0.046	0.28	M	3
***Strongylocentrotus purpuratus***	BCA	H3I177	0.804	0.047	0.16	M	2
***Tetrahymena thermophila***	BCA1	Q22U21	0.092	0.064	0.92	-	1
	BCA2	Q22U16	0.087	0.075	0.918	-	1
	BCA3	I7MDL7	0.659	0.067	0.203	M	3
	BCA4	I7LWM1	0.115	0.058	0.871	-	2
	BCA5	I7M0M0	0.087	0.034	0.947	-	1
	BCA6	I7MD92	0.058	0.069	0.941	-	1
	BCA7	I7M748	0.09	0.047	0.933	-	1
	BCA8	Q23AV1	0.187	0.123	0.758	-	3
***Tribolium castaneum***	BCA	D6WK56	0.054	0.097	0.938	-	1
***Trichinella spiralis***	BCA	E5SH53	0.876	0.028	0.177	M	2
***Trichomonas vaginalis***	BCA1	A2ENQ8	0.043	0.137	0.933	-	2
	BCA2	A2DLG4	0.073	0.061	0.937	-	1
***Trichoplax adhaerens***	BCA	B3S5Y1	0.582	0.038	0.459	M	5
***Xenoturbella bocki***	BCA	117195962	0.222	0.056	0.78	-	3

## Discussion

This study shows that the β-CA enzyme is present in a range of protozoans and metazoans. A total of 75 sequences were identified and a phylogenetic tree constructed. The multiple sequence alignment results revealed that all 75 sequences have the highly conserved residues (Cysteine, Aspartic acid, Arginine, and Histidine) consistent with a β-CA enzyme (Additional file 1: Figure S1). Most of the metazoan and protozoan β-CAs, and corresponding coding sequences, were designated as uncharacterized sequences or CAs with no class specification. These can be now assigned to β-CAs in proteomics and genomics databases.

β-CAs have been identified in the mitochondria of a variety of different organisms, such as plants [61], green algae [62], fungi [1,63], and *Drosophila melanogaster*[4]. Our results of subcellular localization prediction (Table 2) suggested that 31 of the β-CAs are targeted to mitochondria. In mitochondrial targeting peptides (mTPs), Arginine, Alanine and Serine are over-represented, while negatively charged amino acid residues (Aspartic acid and Glutamic acid) are rare. Furthermore, mTPs are believed to form an amphiphilic α-helix, which is important for the import of the nascent protein into the mitochondrion [59]. The successful construction of the TargetP predictor demonstrates that protein sorting signals can be recognized with reasonable reliability from amino acid sequence data alone, thus, to some extent, mimicking the cellular recognition processes [59]. The prediction of the mitochondrial localization for many of the proteins studied is also supported by the previous experimental data, showing that recombinant DmBCA protein is indeed located in mitochondria of insect cells [4]. As mitochondrial proteins the β-CAs may contribute to key metabolic functions. Among the mammalian α-CAs, CA VA and CA VB are the only enzymes that have been exclusively located to mitochondria. Functional studies, summarized in [64], have indicated them in several metabolic processes, such as gluconeogenesis, urea synthesis, and fatty acid synthesis. It has been shown previously that the gluconeogenic enzyme, pyruvate carboxylase, is expressed in protozoan (*Toxoplasma gondii*) mitochondria [65]. This enzyme utilizes bicarbonate to convert pyruvate to oxaloacetate. Mitochondrial CA V is also involved in lipid synthesis through pyruvate carboxylation reaction [66]. Importantly, lipid metabolism is of crucial importance for parasites. Lipids serve as cellular building blocks, signaling molecules, energy stores, posttranslational modifiers, and pathogenesis factors [67]. Parasites rely on complex metabolic systems to satisfy their lipid needs. The present findings open a new avenue to investigate whether mitochondrial β-CAs are functionally involved in these processes.

The single β-CA of *Anopheles darlingi* is the first predicted secretory β-CA. Among the various α-CAs, the first secreted form (CA VI) was identified in human saliva in 1987 [68], and in 2011 another α-CA was identified in the salivary gland of *Aedes aegypti*[69]. Complementary research, such as morphological, biochemical, and spatial mapping of gene expression in *Anopheles darlingi* will clarify the exact expression pattern of β-CA in this mosquito [69,70]*.*

The TargetP predictor defined 43 β-CAs with ‘other’ cellular localizations. Although it is possible that β-CAs are truly located in different subcellular compartments depending on the species, these results should be interpreted with caution. Both the common errors in full genomic DNA, cDNA, or protein sequences in databases, and the potential inaccuracy of TargetP predictor could contribute to the observed deviations of the results. The highest prediction accuracy, with appropriate selection of specificity and sensitivity, is 90% [59].

Among the species mentioned in Table 1, some have important medical relevance, such as *Aedes aegypti, Anopheles darlingi, Anopheles gambiae, Ascaris suum (Ascaris lumbricoides), Culex quinquefasciatus, Entamoeba histolytica, Hirudo medicinalis, Leishmania* species*, Schistosoma mansoni, Trichinella spiralis*, and *Trichomonas vaginalis.* In the past decade, inhibition profiles of β-CAs of bacteria [24,31,71] and fungi [72-75] have been investigated with various inhibitors. Our results suggest that various protozoans and metazoans express β-CAs and that these molecules represent protein targets appropriate for inhibitor development. These proteins are not restricted to nematodes, insects, or protozoa causing human diseases, but are also present in many species with relevance to agriculture or veterinary medicine. These species include: *Acyrthosiphon pisum*, *Ancylostoma caninum*, *Ascaris suum*, *Caligus clemensi*, *Camponotus floridanus*, *Culex quinquefasciatus*, *Dendroctonus ponderosae*, *Entamoeba* species*, Ichthyophthirius multifiliis*, *Solenopsis invicta*, *Tribolium castaneum*, *Trichinella spiralis*, and *Trichoplax adhaerens.* Therefore, our findings also suggest that it might be possible to develop specific β-CA inhibitors as pesticides for the protection of crops and other natural resources against pathogens and pests.

## Conclusions

The present data identifies β-CA enzymes that are expressed in a number of protozoans and metazoans. Metazoan and protozoan β-CAs represent promising diagnostic and therapeutic targets for parasitic infections, because this CA family is absent from mammalian proteomes. Many of these enzymes are predicted to be present in mitochondria where they might contribute to cell metabolism by providing bicarbonate for biosynthetic reactions and regulating intra-mitochondrial pH.

## Competing interests

The authors declare that they have no competing interests.

## Authors’ contributions

RZE, HB, MEET, CO carried out the bioinformatics searches on metazoan and protozoan species. RZE and HB participated in the sequence alignment and made the phylogenetic analysis. RZE performed the mitochondrial targeting peptide prediction. All authors participated in the design of the study. RZE and HB drafted the first version of the manuscript. All authors read and approved the final manuscript.

## Supplementary Material

Additional file 1: Figure S1 Multiple sequence alignment of all 75 β-CAs in metazoan and protozoan species with β-CA of *Pelosinus fermentans* (a bacterial out group). β-CAs contain two highly conserved active site motifs, CxDxR as well as HxxC (C=Cysteine, D=Aspartic acid, R=Arginine, H=Histidine, C=Cysteine) which are indicated by arrows. Alignment was visualized in Jalview [53]. Click here for file
