# Supplementary material for: Bioinformatic analysis of beta carbonic anhydrase sequences from protozoans and metazoans
Source: Parasit Vectors. 2014 Jan 21;7:38. doi: 10.1186/1756-3305-7-38 (PMC3907363; doi:10.1186/1756-3305-7-38)
Supplement: Additional file 1: Figure S1 — Multiple sequence alignment of all 75 β-CAs in metazoan and protozoan species with β-CA of Pelosinus fermentans (a bacterial out group). β-CAs contain two highly conserved active site motifs, CxDxR as well as HxxC (C=Cysteine, D=Aspartic acid, R=Arginine, H=Histidine, C=Cysteine) which are indicated by arrows. Alignment was visualized in Jalview [53]. [file 1756-3305-7-38-S1.pdf]

Supplementary Figure 1. Multiple sequence alignment of all 75 β-CAs in metazoan and protozoan species with β-CA of *Pelosinus fermentans* (a bacterial out group). β-CAs contain two highly conserved active site motifs, CxDxR as well as HxxC (C=Cysteine, D=Aspartic acid, R=Arginine, H=Histidine, C=Cysteine) which are indicated by arrows. Alignment was visualized in Jalview [53].

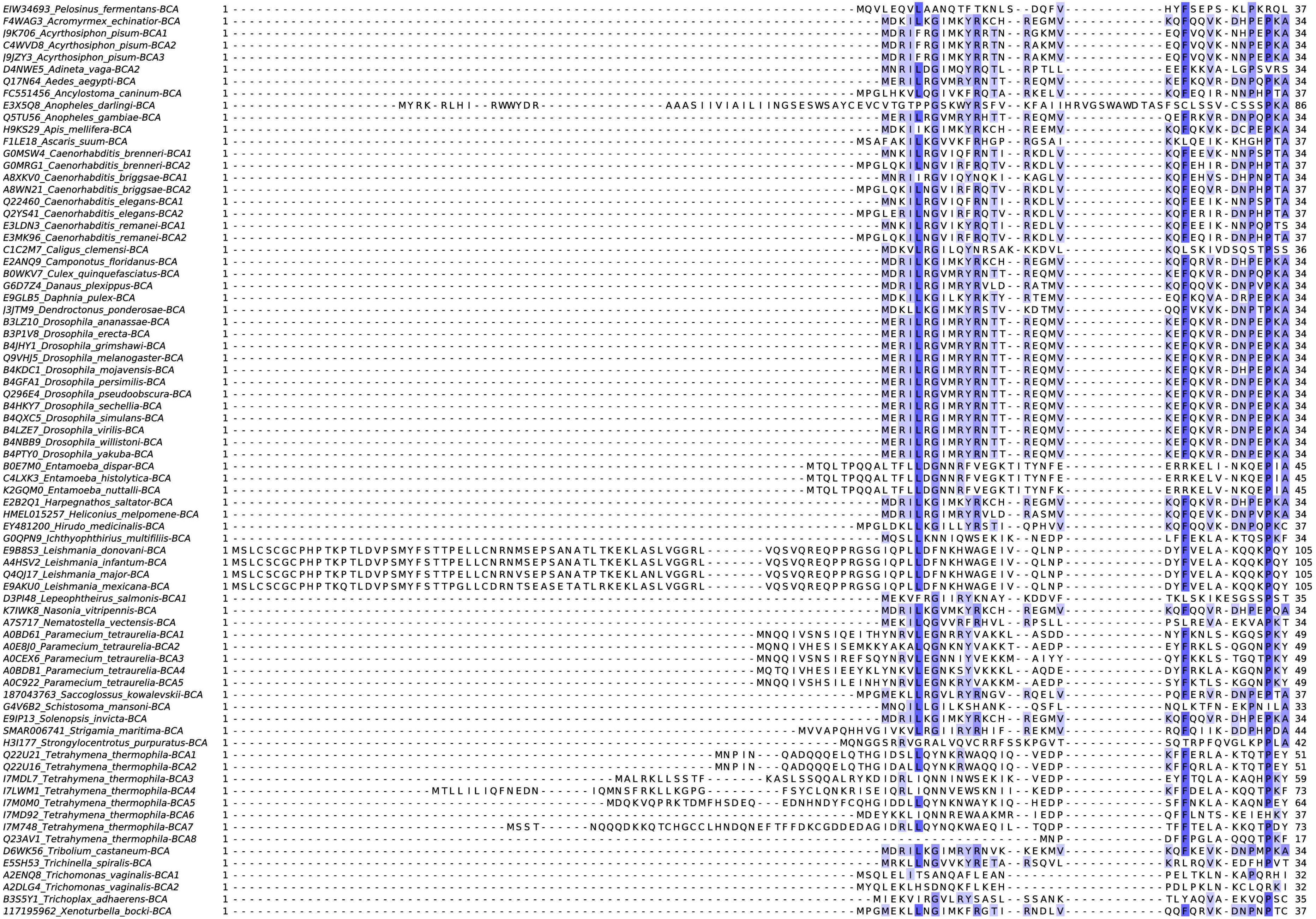
EIW34693_Pelosinus_fermentans-BCA
F4WAG3_Acromyrmex_echinatior-BCA
J9K706_Acyrthosiphon_pisum-BCA1
C4WVD8_Acyrthosiphon_pisum-BCA2
J9JZY3_Acyrthosiphon_pisum-BCA3
D4NWE5_Adineta_vaga-BCA2
Q17N64_Aedes_aegypti-BCA
FC551456_Ancylostoma_caninum-BCA
E3X5Q8_Anopheles_darlingi-BCA
Q5TU56_Anopheles_gambiae-BCA
H9KS29_Apis_mellifera-BCA
F1LE18_Ascaris_suum-BCA
G0MSW4_Caenorhabditis_brenneri-BCA1
G0MRG1_Caenorhabditis_brenneri-BCA2
A8XKV0_Caenorhabditis_briggsae-BCA1
A8WN21_Caenorhabditis_briggsae-BCA2
Q22460_Caenorhabditis_elegans-BCA1
Q2YS41_Caenorhabditis_elegans-BCA2
E3LDN3_Caenorhabditis_remanei-BCA1
E3MK96_Caenorhabditis_remanei-BCA2
C1C2M7_Caligus_clemensi-BCA
E2ANQ9_Camponotus_floridanus-BCA
B0WKV7_Culex_quinquefasciatus-BCA
G6D7Z4_Danaus_plexippus-BCA
E9GLB5_Daphnia_pulex-BCA
J3JTM9_Dendroctonus_ponderosae-BCA
B3LZ10_Drosophila_ananassae-BCA
B3P1V8_Drosophila_erecta-BCA
B4JHY1_Drosophila_grimshawi-BCA
Q9VHJ5_Drosophila_melanogaster-BCA
B4KDC1_Drosophila_mojavensis-BCA
B4GFA1_Drosophila_persimilis-BCA
Q296E4_Drosophila_pseudoobscura-BCA
B4HKY7_Drosophila_sechellia-BCA
B4QXC5_Drosophila_simulans-BCA
B4LZE7_Drosophila_virilis-BCA
B4NBB9_Drosophila_willistoni-BCA
B4PTY0_Drosophila_yakuba-BCA
B0E7M0_Entamoeba_dispar-BCA
C4LXK3_Entamoeba_histolytica-BCA
K2GQM0_Entamoeba_nuttalli-BCA
E2B2Q1_Harpegnathos_saltator-BCA
HMEL015257_Heliconius_melpomene-BCA
EY481200_Hirudo_medicinalis-BCA
G0QPN9_Ichthyophthirius_multifilis-BCA
E9B8S3_Leishmania_donovani-BCA
A4HSV2_Leishmania_infantum-BCA
Q4QJ17_Leishmania_major-BCA
E9AKU0_Leishmania_mexicana-BCA
D3PI48_Lepeophtheirus_salmonis-BCA1
K7IWK8_Nasonia_vitripennis-BCA
A7S717_Nematostella_vectensis-BCA
A0BD61_Paramecium_tetraurelia-BCA1
A0E8J0_Paramecium_tetraurelia-BCA2
A0CEX6_Paramecium_tetraurelia-BCA3
A0BDB1_Paramecium_tetraurelia-BCA4
A0C922_Paramecium_tetraurelia-BCA5
187043763_Saccoglossus_kowalevskii-BCA
G4V6B2_Schistosoma_mansoni-BCA
E9IP13_Solenopsis_invicta-BCA
SMAR006741_Strigamia_maritima-BCA
H3I177_Strongylocentrotus_purpuratus-BCA
Q22U21_Tetrahymena_thermophila-BCA1
Q22U16_Tetrahymena_thermophila-BCA2
I7MDL7_Tetrahymena_thermophila-BCA3
I7LWM1_Tetrahymena_thermophila-BCA4
I7M0M0_Tetrahymena_thermophila-BCA5
I7MD92_Tetrahymena_thermophila-BCA6
I7M748_Tetrahymena_thermophila-BCA7
Q23AV1_Tetrahymena_thermophila-BCA8
D6WK56_Tribolium_castaneum-BCA
E5SH53_Trichinella_spiralis-BCA
A2ENQ8_Trichomonas_vaginalis-BCA1
A2DLG4_Trichomonas_vaginalis-BCA2
B3S5Y1_Trichoplax_adhaerens-BCA
117195962_Xenoturbella_bocki-BCA

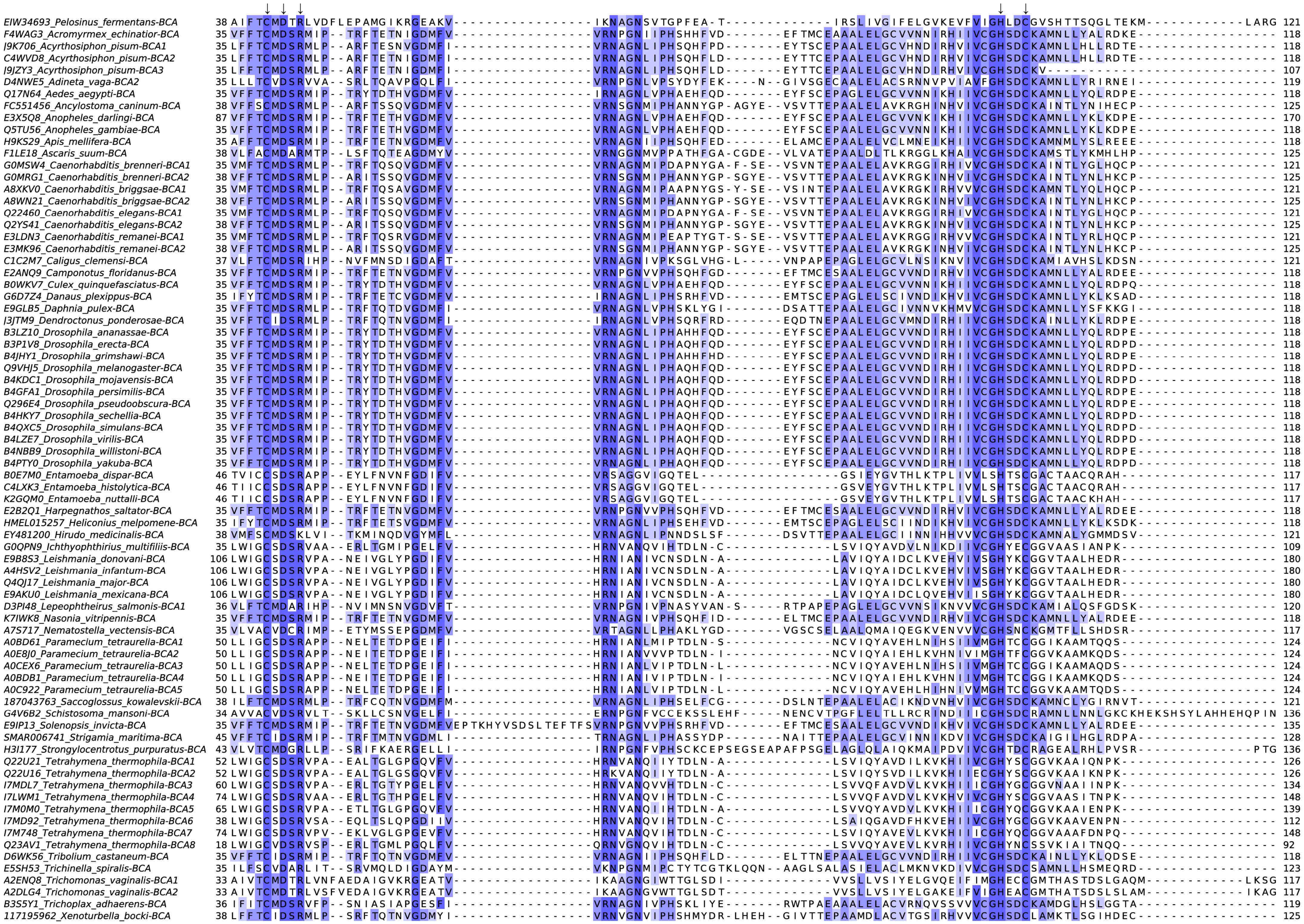

EIW34693_Pelosinus_fermentans-BCA 38 121
F4WAG3_Acromyrmex_echinatior-BCA 35 118
J9K706_Acyrthosiphon_pisum-BCA1 35 118
C4WVD8_Acyrthosiphon_pisum-BCA2 35 118
J9JZY3_Acyrthosiphon_pisum-BCA3 35 107
D4NWE5_Adineta_vaga-BCA2 35 119
Q17N64_Aedes_aegypti-BCA 35 118
FC551456_Ancylostoma_caninum-BCA 38 125
E3X5Q8_Anopheles_darlingi-BCA 87 170
Q5TU56_Anopheles_gambiae-BCA 35 118
H9KS29_Apis_mellifera-BCA 35 118
F1LE18_Ascaris_suum-BCA 38 125
G0MSW4_Caenorhabditis_brenneri-BCA1 35 121
G0MRG1_Caenorhabditis_brenneri-BCA2 38 125
A8XKV0_Caenorhabditis_briggsae-BCA1 35 121
A8WN21_Caenorhabditis_briggsae-BCA2 38 125
Q22460_Caenorhabditis_elegans-BCA1 35 121
Q2YS41_Caenorhabditis_elegans-BCA2 38 125
E3LDN3_Caenorhabditis_remanei-BCA1 35 121
E3MK96_Caenorhabditis_remanei-BCA2 38 125
C1C2M7_Caligus_clemensi-BCA 37 121
E2ANQ9_Camponotus_floridanus-BCA 35 118
B0WKV7_Culex_quinquefasciatus-BCA 35 118
G6D7Z4_Danaus_plexippus-BCA 35 118
E9GLB5_Daphnia_pulex-BCA 35 118
J3JTM9_Dendroctonus_ponderosae-BCA 35 118
B3LZ10_Drosophila_ananassae-BCA 35 118
B3P1V8_Drosophila_erecta-BCA 35 118
B4JHY1_Drosophila_grimshawi-BCA 35 118
Q9VHJ5_Drosophila_melanogaster-BCA 35 118
B4KDC1_Drosophila_mojavensis-BCA 35 118
B4GFA1_Drosophila_persimilis-BCA 35 118
Q296E4_Drosophila_pseudoobscura-BCA 35 118
B4HKY7_Drosophila_sechellia-BCA 35 118
B4QXC5_Drosophila_simulans-BCA 35 118
B4LZE7_Drosophila_virilis-BCA 35 118
B4NBB9_Drosophila_willistoni-BCA 35 118
B4PTY0_Drosophila_yakuba-BCA 35 118
B0E7M0_Entamoeba_dispar-BCA 46 117
C4LXK3_Entamoeba_histolytica-BCA 46 117
K2GQM0_Entamoeba_nuttalli-BCA 46 117
E2B2Q1_Harpegnathos_saltator-BCA 35 118
HMEL015257_Heliconius_melpomene-BCA 35 118
EY481200_Hirudo_medicinalis-BCA 38 121
G0QPN9_Ichthyophthirius_multifiliis-BCA 35 109
E9B8S3_Leishmania_donovani-BCA 106 180
A4HSV2_Leishmania_infantum-BCA 106 180
Q4QJ17_Leishmania_major-BCA 106 180
E9AKU0_Leishmania_mexicana-BCA 106 180
D3PI48_Lepeophtheirus_salmonis-BCA1 36 120
K7IWK8_Nasonia_vitripennis-BCA 35 118
A7S717_Nematostella_vectensis-BCA 35 117
A0BD61_Paramecium_tetraurelia-BCA1 50 124
A0E8J0_Paramecium_tetraurelia-BCA2 50 124
A0CEX6_Paramecium_tetraurelia-BCA3 50 124
A0BDB1_Paramecium_tetraurelia-BCA4 50 124
A0C922_Paramecium_tetraurelia-BCA5 50 124
187043763_Saccoglossus_kowalevskii-BCA 38 121
G4V6B2_Schistosoma_mansoni-BCA 34 136
E9IP13_Solenopsis_invicta-BCA 35 135
SMAR006741_Strigamia_maritima-BCA 45 128
H3I177_Strongylocentrotus_purpuratus-BCA 43 136
Q22U21_Tetrahymena_thermophila-BCA1 52 126
Q22U16_Tetrahymena_thermophila-BCA2 52 126
I7MDL7_Tetrahymena_thermophila-BCA3 60 134
I7LWM1_Tetrahymena_thermophila-BCA4 74 148
I7M0M0_Tetrahymena_thermophila-BCA5 65 139
I7MD92_Tetrahymena_thermophila-BCA6 38 112
I7M748_Tetrahymena_thermophila-BCA7 74 148
Q23AV1_Tetrahymena_thermophila-BCA8 18 92
D6WK56_Tribolium_castaneum-BCA 35 118
E5SH53_Trichinella_spiralis-BCA 35 123
A2ENQ8_Trichomonas_vaginalis-BCA1 33 117
A2DLG4_Trichomonas_vaginalis-BCA2 33 117
B3S5Y1_Trichoplax_adhaerens-BCA 36 119
117195962_Xenoturbella_bocki-BCA 38 125

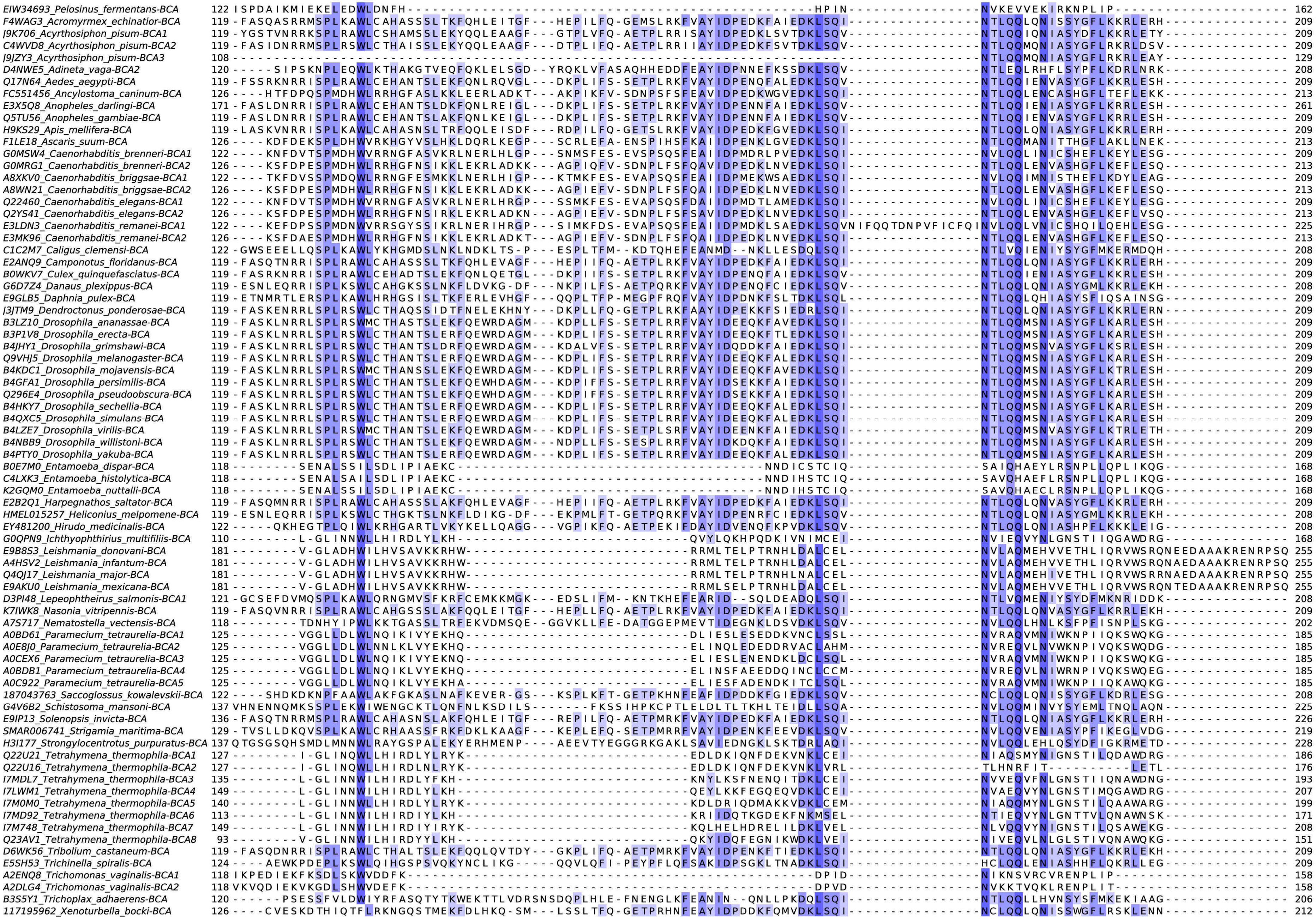

EIW34693_Pelosinus_fermentans-BCA 122 ISPDAIKMIEKELEDWLDNFH---HPIN----------------NVKEVVEKIRKNPLIP--------------------- 162
F4WAG3_Acromyrmex_echinatior-BCA 119 -FASQASRRMSPLKAWLCAHASSSLTKFQHLEITGF---HEPILFQ-GEMSLRKFVAYIDPEDKFAIEDKLSQI----------------NTLQQLQNISSYGFLKKRLERH--------------- 209
J9K706_Acyrthosiphon_pisum-BCA1 119 -YGSTVNRRKSPLRAWLCSHAMSSLEKYQQLEAAGF---GTPLVFQ-AETPLRRISAYIDPEDKLSVTDKLSQV----------------NTLQQIQNIASYDFLKKRLETY--------------- 209
C4WVD8_Acyrthosiphon_pisum-BCA2 119 -FASIDNRRMSPLRSWLCTHAISSLEKYQQLEAAGF---DTPLIFQ-AETPLRRIIAYIDPEDKFSVTDKLSQV----------------NTLQQMQNIASYGFLRKRLDSV--------------- 209
J9JZY3_Acyrthosiphon_pisum-BCA3 108 --NTLQQMQNIASYGFLRKRLEAY--------------- 129
D4NWE5_Adineta_vaga-BCA2 120 -----SIPSKNPLEQWLKTHAKGTVEQFQKLELSGD--YRQKLVFASAQHHEDDFEAYIDPNNEFKSSDKLSQV----------------NTLEQLRHFLSYPFLKDRLNRK--------------- 208
Q17N64_Aedes_aegypti-BCA 119 -FSSRKNRRISPLRAWLCEHANTSLEKFQNLRQVGL---DKPLIFS-SETPLRKFVAYIDPENQFALEDKLSQV----------------NTLQQIENVASYGFLKKRLESH--------------- 209
FC551456_Ancylostoma_caninum-BCA 126 ----HTFDPQSPMDHWLRRHGFASLKKLEERLADKT---AKPIKFV-SDNPSFSFEAVIDPEDKWGVEDKLSQI----------------NTLQQLENCASHGFLTEFLEKK--------------- 213
E3X5Q8_Anopheles_darlingi-BCA 171 -FASLDNRRISPLRAWLCEHANTSLDKFQNLREIGL---DKPLIFS-SETPLRKFVAYIDPENNFAIEDKLSQV----------------NTLQQIENIASYGFLKRRLESH--------------- 261
Q5TU56_Anopheles_gambiae-BCA 119 -FASLDNRRISPLRAWLCEHANTSLAKFQNLKEIGL---DKPLIFS-SETPLRKFVAYIDPENNFAIEDKLSQV----------------NTLQQIENVASYGFLKRRLESH--------------- 209
H9KS29_Apis_mellifera-BCA 119 -LASKVNRRISPLKAWLCAHASNSLTRFQQLEISDF---RDPILFQ-GETSLRKFVAYIDPEDKFGVEDKLSQI----------------NTLQQLQNIASYGFLKKRLERH--------------- 209
F1LE18_Ascaris_suum-BCA 126 ----KDFDEKSPLDHWVRKHGYVSLHKLDQRLKEGP---SCRLEFA-ENSPIHSFKAIIDPENKLGVEDKLSQI----------------NTLQQMANITTHGFLAKLLNEK--------------- 213
G0MSW4_Caenorhabditis_brenneri-BCA1 122 ----KNFDVTSPMDHWVRRNGFASVKRLNERLHLGP---SNMSFES-EVSPSQSFEAIIDPMDRLPVEDKLSQI----------------NVLQQLINICSHEFLKEYLESG--------------- 209
G0MRG1_Caenorhabditis_brenneri-BCA2 126 ----KSFDPESPMDHWLRRHGFNSIKKLEKRLADKK---AGPIQFV-SDNPLFSFQAVIDPEDKLNVEDKLSQI----------------NTLQQLENVASHGFLKEFLESQ--------------- 213
A8XKV0_Caenorhabditis_briggsae-BCA1 122 ----TKFDVSSPMDQWLRRNGFESMKKLNERLHIGP---KTMKFES-EVAPSQSFEAIIDPMEKWSAEDKLSQI----------------NVLQQIMNISTHEFLKDYLEAG--------------- 209
A8WN21_Caenorhabditis_briggsae-BCA2 126 ----KSFDPESPMDHWLRRHGFNSIKKLEKRLADKK---AGPIEFV-SDNPLFSFQAIIDPEDKLNVEDKLSQI----------------NTLQQLENVASHGFLKEFLESQ--------------- 213
Q22460_Caenorhabditis_elegans-BCA1 122 ----KNFDVTSPMDHWVRRNGFASVKRLNERLHRGP---SSMKFES-EVAPSQSFDAIIDPMDTLAMEDKLSQI----------------NVLQQLINICSHEFLKEYLESG--------------- 209
Q2YS41_Caenorhabditis_elegans-BCA2 126 ----KSFDPESPMDHWLRRHGFNSIRKLEKRLADKN---AGPIEFV-SDNPLFSFSAVIDPEDKLNVEDKLSQI----------------NTLQQLENVASHGFLKEFLVSQ--------------- 213
E3LDN3_Caenorhabditis_remanei-BCA1 122 ----KEFDPSSPMDNWVRRSGYSSIKRLNERIHRGP---SIMKFDS-EVAPSQSFEAIIDPMDKLSAEDKLSQVNIFQQTDNPVFICFQINVLQQLVNICSHQILQEHLESG--------------- 225
E3MK96_Caenorhabditis_remanei-BCA2 126 ----KSFDAESPMDHWLRRHGFNSIKKLEKRLADKT---AGPIEFV-SDNPLFSFQAIIDPEDKLNVEDKLSQI----------------NTLQQLENVASHGFLKEFLESQ--------------- 213
C1C2M7_Caligus_clemensi-BCA 122 -GWSEEELLQSPLKAWLYKHGMDSLNKLNDKLTS-P---ESPLTFM--KDTQHEFEANMD--NKLLESDQLSQI----------------NTLVQIENIYSYGFMKERMDQH--------------- 208
E2ANQ9_Camponotus_floridanus-BCA 119 -FASQTNRRISPLRAWLCAHASSSLTKFQHLEVAGF---HEPIIFQ-AETPLRKFVAYIDPEDKFAIEDKLSQI----------------NTLQQLQNIASYGFLKKRLERH--------------- 209
B0WKV7_Culex_quinquefasciatus-BCA 119 -FASRKNRRISPLRAWLCEHADTSLEKFQNLQETGL---DKPIIFS-SETPLRKFVAYIDPENQFAIEDKLSQV----------------NTLQQIENIASYGFLKKRLESH--------------- 209
G6D7Z4_Danaus_plexippus-BCA 119 -ESNLEQRRISPLKSWLCAHGKSSLNKFLDVKG-DF---NKPILFS-AETPQRKFVAYIDPENQFCIEDKLSQV----------------NTLQQLQNIASYGMLKKRLEKH--------------- 208
E9GLB5_Daphnia_pulex-BCA 119 -ETNMRTLERSPLKAWLHRHGSISLTKFERLEVHGF---QQPLTFP-MEGPFRQFVAYIDPDNKFSLTDKLSQL----------------NTLQQLQHIASYSFIQSAINSG--------------- 209
J3JTM9_Dendroctonus_ponderosae-BCA 119 -FASKENRRLSPLRSWLCTHAQSSIDTFNELEKHNY---DKPLLFQ-GETPLRKFAAYIDPEKKFSIEDRLSQI----------------NTLQQLQNIASYGFLKKRLERN--------------- 209
B3LZ10_Drosophila_ananassae-BCA 119 -FASKLNRRLSPLRSWMCTHASTSLEKFQEWRDAGM---KDPLLFS-SETPLRRFVAYIDEEQKFAVEDKLSQI----------------NTLQQMSNIASYGFLKARLESH--------------- 209
B3P1V8_Drosophila_erecta-BCA 119 -FASKLNRRLSPLRSWLCTHANTSLERFQEWRDAGM---KDPLIFS-SETPLRRFVAYIDEEQKFTLEDKLSQI----------------NTLQQMSNIASYGFLKARLESH--------------- 209
B4JHY1_Drosophila_grimshawi-BCA 119 -FASKLNRRLSPLRSWLCTHANTSLDRFQEWRDAGM---KDALVFS-SETPLRRFVAYIDQDDKFAIEDKLSQI----------------NTLQQMSNVASYGFLKSRLESH--------------- 209
Q9VHJ5_Drosophila_melanogaster-BCA 119 -FASKLNRRLSPLRSWLCTHANTSLERFQEWRDAGM---KDPLIFS-SETPLRRFVAYIDEEQKFALEDKLSQI----------------NTLQQMSNIASYGFLKARLESH--------------- 209
B4KDC1_Drosophila_mojavensis-BCA 119 -FASKLNRRLSPLRSWMCTHANSSLEKFQEWRDAGM---KDPLIFS-SETPLRRFVAYIDEEQKFAVEDKLSQI----------------NTLQQMSNIASYGFLKTRLESH--------------- 209
B4GFA1_Drosophila_persimilis-BCA 119 -FASKLNRRLSPLRSWLCTHANTSLEKFQEWHDAGM---KDPIFFS-SETPLRRFVAYIDSEKKFAIEDKLSQI----------------NTLQQMSNIASYGFLKARLESH--------------- 209
Q296E4_Drosophila_pseudoobscura-BCA 119 -FASKLNRRLSPLRSWLCTHANTSLEKFQEWHDAGM---KDPIFFS-SETPLRRFVAYIDSEKKFAIEDKLSQI----------------NTLQQMSNIASYGFLKARLESH--------------- 209
B4HKY7_Drosophila_sechellia-BCA 119 -FASKLNRRLSPLRSWLCTHANTSLERFQEWRDAGM---KDPLIFS-SETPLRRFVAYIDEEQKFALEDKLSQI----------------NTLQQMSNIASYGFLKARLESH--------------- 209
B4QXC5_Drosophila_simulans-BCA 119 -FASKLNRRLSPLRSWLCTHANTSLERFQEWRDAGM---KDPLIFS-SETPLRRFVAYIDEEQKFALEDKLSQI----------------NTLQQMSNIASYGFLKARLESH--------------- 209
B4LZE7_Drosophila_virilis-BCA 119 -FASKLNRRLSPLRSWMCTHANTSLEKFQEWRDAGM---KDPLIFS-SETPLSRFVAYIDEENKFAIEDKLSQI----------------NTLQQMSNVASYGFLKTRLETH--------------- 209
B4NBB9_Drosophila_willistoni-BCA 119 -FASKLNRRLSPLRSWLCTHANTSLEKFQEWRDAGM---NDPLLFS-SESPLRRFVAYIDKDQKFAIEDKLSQI----------------NTLQQMSNIASYGFLKARLESH--------------- 209
B4PTY0_Drosophila_yakuba-BCA 119 -FASKLNRRLSPLRSWLCTHANTSLERFQEWRDAGM---KDPLIFS-SETPLRRFVAYIDEEQKFALEDKLSQI----------------NTLQQMSNIASYGFLKARLESH--------------- 209
B0E7M0_Entamoeba_dispar-BCA 118 --------SENALSSILSDLIPIAEKC-------------------------------------NNDICSTCIQ----------------SAIQHAEYLRSNPLLQPLIKQG--------------- 168
C4LXK3_Entamoeba_histolytica-BCA 118 --------SENALSAILSDLIPIAEKC-------------------------------------NNDIHSTCIQ----------------SAVQHAEFLRSNPLLQPLIKQG--------------- 168
K2GQM0_Entamoeba_nuttalli-BCA 118 --------SENALSSILSDLIPIAEKC-------------------------------------NNDIHSTCIQ----------------SAVQHAECLRSNPLLQPLIKQG--------------- 168
E2B2Q1_Harpegnathos_saltator-BCA 119 -FASQMNRRISPLRAWLCAHASSSLAKFQHLEVAGF---HEPIIFQ-AETPLRKFVAYIDPEDKFAIEDKLSQI----------------NTLQQLQNVASYGFLKKRLERH--------------- 209
HMEL015257_Heliconius_melpomene-BCA 119 -ESNLEQRRISPLKSWLCTHGKTSLNKFLDIKG-DF---EKPMLFT-GETPQRKFVAYIDPENRFCIEDKLSQV----------------NTLQQLQNIASYGMLKKRLEKH--------------- 208
EY481200_Hirudo_medicinalis-BCA 122 -----QKHEGTPLQIWLKRHGARTLVKYKELLQAGG---VGPIKFQ-AETPEKIFDAYIDVENQFKPVDKLSQV----------------NTLQQLQNIASHPFLKKKLEIG--------------- 208
G0QPN9_Ichthyophthirius_multifiliis-BCA 110 --------L-GLINNWLLHIRDLYLKH----------------------------QVYLQKHPQDKIVNIMCEI----------------NVIEQVYNLGNSTIIQGAWDRG--------------- 168
E9B8S3_Leishmania_donovani-BCA 181 --------V-GLADHWILHVSAVKKRHW---------------------------RRMLTELPTRNHLDALCEL----------------NVLAQMEHVVETHLIQRVWSRQNEEDAAAKRENRPSQ 255
A4HSV2_Leishmania_infantum-BCA 181 --------V-GLADHWILHVSAVKKRHW---------------------------RRMLTELPTRNHLDALCEL----------------NVLAQMEHVVETHLIQRVWSRQNAEDAAAKRENRPSQ 255
Q4QJ17_Leishmania_major-BCA 181 --------V-GLADHWILHVSAVKKRHW---------------------------RRMLTELPTRNHLNALCEL----------------NVLAQMEHIVETHLIQRVWSRRNAEDAAAKRENRPSQ 255
E9AKU0_Leishmania_mexicana-BCA 181 --------V-GLADHWILHVSAVKKRHW---------------------------RRMLSELPTRNHLDALCEL----------------NVLAQMEHVVETHLIQRVWSRQNTEDAAAKRENRPSQ 255
D3PI48_Lepeophtheirus_salmonis-BCA1 121 -GCSEFDVMQSPLKAWLQRNGMVSFKRFCEMKKMGK---EDSLIFM--KNTKHEFEARID--SQLDEADQLSQI----------------NTLVQMENIYSYDFMKNRIDDK--------------- 208
K7IWK8_Nasonia_vitripennis-BCA 119 -FASQVNRRISPLRAWLCAHGSSSLAKFQQLEITGF---HEPLLFQ-AETPLRKFVAYIDPEDKFAIEDKLSQI----------------NTLQQLQNVASYGFLKRRLEKH--------------- 209
A7S717_Nematostella_vectensis-BCA 118 --------TDNHYIPWLKKTGASSLTRFEKVDMSQE--GGVKLLFE-DATGGEPMEVTIDEGNKLDSVDKLSQV----------------NVLQQLHNLKSFPFISNPLSKG--------------- 202
A0BD61_Paramecium_tetraurelia-BCA1 125 --------VGGLLDLWLNQIKIVYEKHQ---------------------------DLIESLESEDDKVNCLSSL----------------NVRAQVMNIWKNPIIQKSWQKG--------------- 185
A0E8J0_Paramecium_tetraurelia-BCA2 125 --------VGGLLDLWLNNLKLVYEKHQ---------------------------ELINQLEDEDDRVACLAHM----------------NVREQVLNVWKNPIVQKSWQDG--------------- 185
A0CEX6_Paramecium_tetraurelia-BCA3 125 --------VGGLLDLWLNQIKIVYEKNQ---------------------------ELIESLENENDKLDCLSQL----------------NVRAQVMNIWKNPIIQKSWQKG--------------- 185
A0BDB1_Paramecium_tetraurelia-BCA4 125 --------VGGLLDLWLNQIKLVYEKHQ---------------------------ELINSFAEEDDQINCLCCM----------------NVREQVLNIWRNPIVQKSWQEG--------------- 185
A0C922_Paramecium_tetraurelia-BCA5 125 --------VGGLLDLWLNQIKLVYEKHA---------------------------ELIESFADENDKITCLSQL----------------NVRAQVMNIWKNPIIQKAWQKG--------------- 185
187043763_Saccoglossus_kowalevskii-BCA 122 ----SHDKDKNPFAAWLAKFGKASLNAFKEVER-GS---KSPLKFT-GETPKHNFEAFIDPDDKFGIEDKLSQV----------------NCLQQLQNISSYGFLKDRLESG--------------- 208
G4V6B2_Schistosoma_mansoni-BCA 137 VHNENNQMKSSPLEKWIWENGCKTLQNFNLKSDILS-------FKSSIHPKCPTLELDLTLTKHLTEIDLLSQA----------------NVLQQMINVYSYEMLTNQLAQN--------------- 225
E9IP13_Solenopsis_invicta-BCA 136 -FASQTNRRMSPLRAWLCAHASNSLAKFQHLEITGF---REPILFQ-AETPMRKFVAYIDPEDKFAIEDKLSQI----------------NTLQQLQNIASYGFLKKRLERH--------------- 226
SMAR006741_Strigamia_maritima-BCA 129 -TVSLLDKQVSPLKAWLCRHAASSFRKFDKLKAAGF---KEPLEFQ-SETPMRRFVAYIDPENKFEEVDKLSQV----------------NVLQQVENIASYPFIKEGLVDG--------------- 219
H3I177_Strongylocentrotus_purpuratus-BCA 137 QTGSGSQHSMDLMNNWLRAYGSPALEKYERHMENP----AEEVTYEGGGRKGAKLSAVIEDNGKLSKTDRLAQI----------------NVLQQLEHLQSYDFIGKRMETD--------------- 228
Q22U21_Tetrahymena_thermophila-BCA1 127 --------I-GLINQWLLHIRDLYLRYK---------------------------EDLDKIQNFDEKVNKLCEI----------------NIAQSMYNIGNSTILQDAWDRG--------------- 186
Q22U16_Tetrahymena_thermophila-BCA2 127 --------I-GLINQWLLHIRDLNLRYK---------------------------EDLDKIQNFDEKVNKLVRL----------------TLHNRFIT----------LETL--------------- 176
I7MDL7_Tetrahymena_thermophila-BCA3 135 --------L-GLINNWILHIRDLYFKH----------------------------KNYLKSFNENQITDKLCEI----------------NVVEQVFNLGNSTIIQNAWDNG--------------- 193
I7LWM1_Tetrahymena_thermophila-BCA4 149 --------L-GLINNWILHIRDLYLKH----------------------------QEYLKKFEGQEQVDKLCEI----------------NVAEQVYNLGNSTIMQGAWDRG--------------- 207
I7M0M0_Tetrahymena_thermophila-BCA5 140 --------L-GLINNWLLHIRDLYLRYK---------------------------KDLDRIQDMAKKVDKLCEM----------------NIAQQMYNLGNSTILQAAWARG--------------- 199
I7MD92_Tetrahymena_thermophila-BCA6 113 --------L-GLINNWILHIRDIYLKH----------------------------KRIIDQTKGDEKFNKMSEL----------------NTIEQVYNLGNTTVLQNAWNSK--------------- 171
I7M748_Tetrahymena_thermophila-BCA7 149 --------L-GLINNWILHIRDIYIRYK---------------------------KQLHELHDRELILDKLVEL----------------NLVQQVYNIGNSTILQSAWEKG--------------- 208
Q23AV1_Tetrahymena_thermophila-BCA8 93 --------V-GLINNWILHIRDYYLKH----------------------------QKYIDQFEGNIKWDKLVEI----------------NIVEQVLNLGLSTIVQNAWQKG--------------- 151
D6WK56_Tribolium_castaneum-BCA 119 -FASQDNRRISPLRAWLCTHALTSLEKFQQLQVTDY---GKPLIFQ-AETPMRKFVAYIDPENKFTIEDKLSQI----------------NTLQQLQNIASYGFLKKRLEKH--------------- 209
E5SH53_Trichinella_spiralis-BCA 124 ----AEWKPDEPLKSWLQIHGSPSVQKYNCLIKG-----QQVLQFI-PEYPFLQFSAKIDPSGKLTNADKLSQI----------------HCLQQLENIASHHFLQKRLLEG--------------- 209
A2ENQ8_Trichomonas_vaginalis-BCA1 118 IKPEDIEKFKSDLSKWVDDFK---DPID----------------NIKNSVRCVRENPLIP--------------------- 158
A2DLG4_Trichomonas_vaginalis-BCA2 118 VKVQDIEKVKGDLSHWVDEFK---DPVD----------------NVKKTVQKLRENPLIT--------------------- 158
B3S5Y1_Trichoplax_adhaerens-BCA 120 ------PSESSFVLDWIYRFASQTYTKWEKTTLVDRSNSDQPLHLE-FNENGLKFEANIN--QNLLPKDQLSQI----------------NTLQQLLHVNSYSFMKEKIAAG--------------- 206
117195962_Xenoturbella_bocki-BCA 126 ----CVESKDTHIQTFLRKNGQSTMEKFDLHKQ-SM---LSSLTFQ-GETPRHNFEAYIDPDDKFQMVDKLSQI----------------NCLQQLQNISSWGFLRSKLENN--------------- 212

```
EIW34693_Pelosinus_fermentans-BCA           163 ----KDVPIHGLMFNPHTGELEVVVNGY---------------------------------------------------------------------------------------------- 186
F4WAG3_Acromyrmex_echinatior-BCA            210 -----DLHIHALWFDIYTGDIYYFSRANKRF-----IEINETTEQLLLKEINKY-YS----------------------------------------------------------------- 255
J9K706_Acyrthosiphon_pisum-BCA1             210 -----DLHIHALWFDIYTGDVHYFSRQSKQF-----VEINEKNVDGLVEEVSKY-YC----------------------------------------------------------------- 255
C4WVD8_Acyrthosiphon_pisum-BCA2                 --------------------------------------------------------------------------------------------------------------------------
J9JZY3_Acyrthosiphon_pisum-BCA3             130 -----DLHIHALWFDIYTGDIYYFSRQSKKF-----VEINEKNVDKLVEEVSKY-YC----------------------------------------------------------------- 175
D4NWE5_Adineta_vaga-BCA2                    209 -----ELQVHALWTDIYKGEVYMFSFKEKCF-----IKIDENTYKKLSFECTPN-K------------------------------------------------------------------ 253
Q17N64_Aedes_aegypti-BCA                    210 -----DLHIHALWFDIYTGDIYYFSRNSKRF-----IPIDETSIEQLLDEVRRY-YS----------------------------------------------------------------- 255
FC551456_Ancylostoma_caninum-BCA            214 -----TVDLHAMWFDIFAGEMYLFSKPRRKF-----ILIDEGTVDKLEEEVNQH-KA----------------------------------------------------------------- 259
E3X5Q8_Anopheles_darlingi-BCA               262 -----DLHIHALWFDIYTGDIYYFSRNSKRF-----ITIDESTIEQLLTEVRRY-YS----------------------------------------------------------------- 307
Q5TU56_Anopheles_gambiae-BCA                210 -----DLHIHALWFDIYTGDIYFFSRNSKRF-----IAIDESSIDRLLDEVRRY-YS----------------------------------------------------------------- 255
H9KS29_Apis_mellifera-BCA                   210 -----DLHIHALWFDI---------------------------------------------------------------------------------------------------------- 220
F1LE18_Ascaris_suum-BCA                     214 -----QADLHAFWFQIETAEMHIFSHKQHKF-----VVINDNTVDELVDEVEHH-TA----------------------------------------------------------------- 259
G0MSW4_Caenorhabditis_brenneri-BCA1         210 -----RLHIHGMWFDIYKGEDYLFSKDKKRF-----VVIDEKTVTDLLAELNTR-YPLPEDQDGPVAFAQSN-------------------------------------------------- 270
G0MRG1_Caenorhabditis_brenneri-BCA2         214 -----TVDLHAMWFDIYTGEMHMFSKPNKRF-----VLVDESNVEELIEEVEKH-QA----------------------------------------------------------------- 259
A8XKV0_Caenorhabditis_briggsae-BCA1         210 -----NLHLHGAWFNIYDGEVFLFSKDRKRF-----VVIDEKTVPSLSAELERR-CPLPEDKAGDVVIQNLH-------------------------------------------------- 270
A8WN21_Caenorhabditis_briggsae-BCA2         214 -----TVDLHAMWFDIYTGEMHMFSKPNNRF-----VLIEESNVEELIDEVEKH-QT----------------------------------------------------------------- 259
Q22460_Caenorhabditis_elegans-BCA1          210 -----RLHIHGMWFDIYKGEDYLFSKDKKRF-----VVIDEKTVTDLLAELNAR-YPVPEDQDGPVAFAKSN-------------------------------------------------- 270
Q2YS41_Caenorhabditis_elegans-BCA2          214 -----TVDLHAMWFDIYTGEMHMFSKPNKQF-----VLVDESNVEELIDEVEKH-QT----------------------------------------------------------------- 259
E3LDN3_Caenorhabditis_remanei-BCA1          226 -----RLHIHGMWFDVYTGDDYLFSKDKKRF-----VVIDEKTVDKHLSELNAR-CPLPEDQDGPVAFAKAK-------------------------------------------------- 286
E3MK96_Caenorhabditis_remanei-BCA2          214 -----TVDLHAMWFDIYTGEMHMFSKPNNRF-----VLVDESNVEELIDEVEKH-QT----------------------------------------------------------------- 259
C1C2M7_Caligus_clemensi-BCA                 209 -----QSVAHGLWLSLSSGEAHFFSKKDKAF-----VNVTEDNVEELVCR------------------------------------------------------------------------ 248
E2ANQ9_Camponotus_floridanus-BCA            210 -----DLHIHALWFDIYTGDIYYFSRANKRF-----VEINEITEPFLLKEIKRY-YS----------------------------------------------------------------- 255
B0WKV7_Culex_quinquefasciatus-BCA           210 -----DLHIHALWFDIYTGDIYYFSRNSKRF-----IPVDETTIERLLKEVNQF-YS----------------------------------------------------------------- 255
G6D7Z4_Danaus_plexippus-BCA                 209 -----DLHIHALWFDIYTGDIYYFSRRAKRF-----LIIDEASYEVILAEIRRY-YS----------------------------------------------------------------- 254
E9GLB5_Daphnia_pulex-BCA                    210 -----RVHLHALWFDIYTGDIYVFSRKQKRF-----VEISEETSDYLLDEIREY-FV----------------------------------------------------------------- 255
J3JTM9_Dendroctonus_ponderosae-BCA          210 -----QLHIHALWFDIYTGEIYYFSRGAKRF-----VIIDESNFEKLTEEVVLY-YS----------------------------------------------------------------- 255
B3LZ10_Drosophila_ananassae-BCA             210 -----DLHIHALWFDIYTGDIYYFSRGAKRF-----VAVDEKSVEELSAEIRRF-YS----------------------------------------------------------------- 255
B3P1V8_Drosophila_erecta-BCA                210 -----DLHIHALWFDIYTGDIYYFSRGAKRF-----LPVDEDTVDRLSEEVRRF-YS----------------------------------------------------------------- 255
B4JHY1_Drosophila_grimshawi-BCA             210 -----NLHIHALWFDIYTGDIYYFSRGAKRF-----IAVDESSVEQLSAEVRRF-YS----------------------------------------------------------------- 255
Q9VHJ5_Drosophila_melanogaster-BCA          210 -----DLHIHALWFDIYTGDIYYFSRGAKRF-----LPVDEDTVDRLSEEVRRF-YS----------------------------------------------------------------- 255
B4KDC1_Drosophila_mojavensis-BCA            210 -----NLHIHALWFDIYTGDIYYFSRGAKRF-----VAVDESSVDQLSAEVRRF-YS----------------------------------------------------------------- 255
B4GFA1_Drosophila_persimilis-BCA            210 -----DLHIHALWFDIYTGDIYYFSRGAKQF-----IAVDEDSVDRLSAEVRRF-YS----------------------------------------------------------------- 255
Q296E4_Drosophila_pseudoobscura-BCA         210 -----DLHIHALWFDIYTGDIYYFSRGAKQF-----IAVDEDSVDRLSAEVRRF-YS----------------------------------------------------------------- 255
B4HKY7_Drosophila_sechellia-BCA             210 -----DLHIHALWFDIYTGDIYYFSRGAKRF-----LPVDEDTVDRLSEEVRRF-YS----------------------------------------------------------------- 255
B4QXC5_Drosophila_simulans-BCA              210 -----DLHIHALWFDIYTGDIYYFSRGAKRF-----LPVDEDTVDRLSEEVRRF-YS----------------------------------------------------------------- 255
B4LZE7_Drosophila_virilis-BCA               210 -----NLHVHALWFDIYTGDIYYFSRGAKRF-----IAVDESSVDQLSAEVRRF-YS----------------------------------------------------------------- 255
B4NBB9_Drosophila_willistoni-BCA            210 -----DLHIHALWFDIYTGDIYYFSRGAKCF-----VAVDEDSVERLSKEVRQF-YS----------------------------------------------------------------- 255
B4PTY0_Drosophila_yakuba-BCA                210 -----DLHIHALWFDIYTGDIYYFSRGAKRF-----LPVDEDTVDRLSEEVRRF-YS----------------------------------------------------------------- 255
B0E7M0_Entamoeba_dispar-BCA                 169 -----QCNIVSMIYNIETGKCEVVQ------------------------------------------------------------------------------------------------- 188
C4LXK3_Entamoeba_histolytica-BCA            169 -----QCNIVSMIYNIETGKCEVVQ------------------------------------------------------------------------------------------------- 188
K2GQM0_Entamoeba_nuttalli-BCA               169 -----QCNIVSMIYNIETGKCEVVQ------------------------------------------------------------------------------------------------- 188
E2B2Q1_Harpegnathos_saltator-BCA            210 -----DLHIHALWFDIYTGDIYYFSRANKRF-----VEINEATEPALLKEIKKY-YS----------------------------------------------------------------- 255
HMEL015257_Heliconius_melpomene-BCA         209 -----DLHIHALWFDIYTGDIYYFSRRAKRF-----IIIDESSYEVILAEIRRY-YS----------------------------------------------------------------- 254
EY481200_Hirudo_medicinalis-BCA             209 -----KVRLHALWIDVYTGDFHMFCRDSNRF-----MLVNEESYENLLADGESN-IDY---------------------------------------------------------------- 255
G0QPN9_Ichthyophthirius_multifiliis-BCA     169 ----QVVNIHGWIYGVNDGRLRDLAITCSNL--KDLTTKHQKYLDFLK-------------------------------------------------------------------------- 210
E9B8S3_Leishmania_donovani-BCA              256 NKPENEVEIHGWVYGLEDGLIRPLLTLNRRS--NAEKELHNAADALFWRY----GQL----------------------------------------------------------------- 306
A4HSV2_Leishmania_infantum-BCA              256 NKPENEVEIHGWVYGLEDGLIRPLLTLNRRS--NAEKELHNAADALFWRY----GQL----------------------------------------------------------------- 306
Q4QJ17_Leishmania_major-BCA                 256 NKPENEVEIHGWVYGLEDGLIRPLLTLNRRS--NAEKELHNAADALFWRY----GQL----------------------------------------------------------------- 306
E9AKU0_Leishmania_mexicana-BCA              256 NKPENEVEIHGWVYGLEDGLIRPLLTLNRRS--NVEKELHNAVDALFWRY----GQL----------------------------------------------------------------- 306
D3PI48_Lepeophtheirus_salmonis-BCA1         209 -----TAFVHGLWFSLTTGDVHYFLKNEKVF-----INVSEDNIDNLVQRSEE--------------------------------------------------------------------- 251
K7IWK8_Nasonia_vitripennis-BCA              210 -----NLHVHALWFDIYTGDIYYFSRANKRF-----VEINELTETPLLKEIKKY-YS----------------------------------------------------------------- 255
A7S717_Nematostella_vectensis-BCA           203 -----ALNLYGLWFDIKEGEMYMFSRKQKKF-----VLINKDTVNNLCSEVD---------------------------------------------------------------------- 244
A0BD61_Paramecium_tetraurelia-BCA1          186 ----NPIMVHGWLFRVETGYIEELLIDSQTP--EEMSKVFALKFKLDSERI----LS---KKASRQSSPQNSTRKRFQLMQKKITQNLKKLKENGGRLEQDIELQLLGELIQNSQQS----- 289
A0E8J0_Paramecium_tetraurelia-BCA2          186 ----HPVMVHGWLFRVETGFIEELEIEESIP--TNLSKVFQLNFKPTQTHK----PKQQKDDNEEERSRSPSIRKRFQRMQSRLETEIKRLTITLQDPNQEVEKDVIDQVSEVLQKDLGIQN 297
A0CEX6_Paramecium_tetraurelia-BCA3          186 ----NPIMVHGWLFRVETGYIEELLIDSQTP--EEMNKVFALKFKLDSERI----LS---KQTTPLGSPKNNARKKFQSMQKQIIQHIKKQKENAGGFQKTNDLQQIREYVQNCQEL----- 289
A0BDB1_Paramecium_tetraurelia-BCA4          186 ----HPVMVHGWLFRVETGFIEELSLEDSIP--EEMSKIFKIKFKPPKVQS----PSQHEDE-EGNAISPMRARRRFQRMQSRIETGIRHFTIHMQNVDEEEIE---HKLVENIEQDLSV-- 291
A0C922_Paramecium_tetraurelia-BCA5          186 ----NPVMVHGWLFRVETGYIEELLIDQHTP--EEMCKVYALKFKLESEKQ----LS---KHVSPTGSPKNNAKKRFQNMQRKLIEHINQLKENKIE-DFDLETKEIGGFIESSKDL----- 288
187043763_Saccoglossus_kowalevskii-BCA      209 -----RVRLHAMWV------------------------------------------------------------------------------------------------------------ 217
G4V6B2_Schistosoma_mansoni-BCA              226 -----VLRVHGMWFEFYSERF----------------------------------------------------------------------------------------------------- 241
E9IP13_Solenopsis_invicta-BCA               227 -----DLHIHALWFDIYTGDIYYFSRANKRF-----VEINETTEPLLLKEIKQY-YS----------------------------------------------------------------- 272
SMAR006741_Strigamia_maritima-BCA           220 -----SIHIHAFWFDIYTGDLYYFSRQQKRF-----ININEKNINKLIMEVG---------------------------------------------------------------------- 261
H3I177_Strongylocentrotus_purpuratus-BCA    229 -----QIRLHATFYDTFSGNVYVFNQKQGRF-----NFLPTADIDSLSHYIFQLRSS----------------------------------------------------------------- 275
Q22U21_Tetrahymena_thermophila-BCA1         187 ----QDISINAWIYSLENGCITELDYAASSR--EVFEEVNEKAIKTILQGI----TSLPSYS-EIVRQKRSSTVPFRKSDFSNDGKQV---------------------------------- 263
Q22U16_Tetrahymena_thermophila-BCA2         177 ----QYYKIHGIEDKIYQQMLGFIVQKMVVLLNQTMQQVQEKFLKKLIKMQ----LR----L-FSKEQPVFRAIQKLKSDFSNDGKQV---------------------------------- 251
I7MDL7_Tetrahymena_thermophila-BCA3         194 ----QSLYIHGWIYGIDNGRVNDLNLSAGNL--TDLEEKYHNYFQQIYKSY----QPKKIQESSQYY------------------------------------------------------- 250
I7LWM1_Tetrahymena_thermophila-BCA4         208 ----QQVYIHGWIYGVKDGRVTDLDLQACNS--EELVKKYYSYINKIKNLF----TVNPHQLKSIPPSPGFKLDWGYKPQYKQTPENLQKKPQ------LMRVDTNNNKFM----------- 302
I7M0M0_Tetrahymena_thermophila-BCA5         200 ----QEITINAWIYSLKDGTITELDYAASSR--EVFEEVQERALKNIIKNI----PDTDEVT-GRLTKRQSSIVIRNSDQFH---------------------------------------- 270
I7MD92_Tetrahymena_thermophila-BCA6         172 ----QKIQIHGWVYGLEDGRIKDLNISSSNY--RELVKNYKRAIKYLKEQ------------------------------------------------------------------------ 215
I7M748_Tetrahymena_thermophila-BCA7         209 ----QEVTIHGWIYGLKDGRVKEIDYAANSK--QVFEEVHDKVISRLLSQI----SS-------------QAKHKLYQDIFQEKTSNSD--SS------LTETKDSINECC----------- 288
Q23AV1_Tetrahymena_thermophila-BCA8         152 ----QLVYIHGWVYGVQDGLVRDLNISSCSL--QQ--------------------------------------------------------------------------------------- 180
D6WK56_Tribolium_castaneum-BCA              210 -----QLHIHALWFDIYTGEIYYFSRGAKKF-----VVIDEENFPKLLQEVEKY-YS----------------------------------------------------------------- 255
E5SH53_Trichinella_spiralis-BCA             210 -----LAKLISEKHKVEDGILLITNENS-SQ-----CPLNTANLLYQGDSITLHVYGLTESVPYWR-------------------------------------------------------- 264
A2ENQ8_Trichomonas_vaginalis-BCA1           159 ----KNIPIHGLLIHPDTGKVTTIINGY---------------------------------------------------------------------------------------------- 182
A2DLG4_Trichomonas_vaginalis-BCA2           159 ----KNIPIHGLVINPDTGKLTTIVNGYNK-------------------------------------------------------------------------------------------- 184
B3S5Y1_Trichoplax_adhaerens-BCA             207 -----TVKLYSLWFDIKDATCYVFNKRDKLF-----QPIVSEEMLKSVSEFQIN-QK----------------------------------------------------------------- 252
117195962_Xenoturbella_bocki-BCA            213 -----EVRLHAMWFDIYTGDHYMFQQNTGNG-----LSKSAKKTWSTY-------------------------------------------------------------------------- 250
```
